# Expressing and Amplifying Positive Emotions Facilitate Goal Attainment in Workplace Interactions

**DOI:** 10.3389/fpsyg.2013.00188

**Published:** 2013-05-09

**Authors:** Elena Wong, Franziska Tschan, Laurence Messerli, Norbert K. Semmer

**Affiliations:** ^1^Institute of Work and Organizational Psychology, Université de NeuchâtelNeuchâtel, Switzerland; ^2^National Center of Competence in Research Affective SciencesNeuchâtel, Switzerland; ^3^Department of Psychology, University of BernBern, Switzerland

**Keywords:** positive emotion, emotion regulation, goals, social interactions at work, superior, coworker, organizations

## Abstract

Expressing emotions has social functions; it provides information, affects social interactions, and shapes relationships with others. Expressing positive emotions could be a strategic tool for improving goal attainment during social interactions at work. Such effects have been found in research on social contagion, impression management, and emotion work. However, expressing emotions one does not feel entails the risk of being perceived as inauthentic. This risk may well be worth taking when the emotions felt are negative, as expressing negative emotions usually has negative effects. When experiencing positive emotions, however, expressing them authentically promises benefits, and the advantage of amplifying them is not so obvious. We postulated that expressing, and amplifying, positive emotions would foster goal attainment in social interactions at work, particularly when dealing with superiors. Analyses are based on 494 interactions involving the pursuit of a goal by 113 employes. Multilevel analyses, including polynomial analyses, show that authentic display of positive emotions supported goal attainment throughout. However, amplifying felt positive emotions promoted goal attainment only in interactions with superiors, but not with colleagues. Results are discussed with regard to the importance of hierarchy for detecting, and interpreting, signs of strategic display of positive emotions.

## Introduction

If an employe pursues a specific goal in an encounter with his or her superior, will the expression of emotions make a difference for goal attainment? Specifically, will expressing *positive* emotions help goal attainment in this situation? If the employe feels slightly positive, is amplifying the expression of these feelings useful for reaching the goal? Would such a strategy also work in interactions with colleagues? In this paper, we investigate whether (a) the expression and (b) the amplification of positive emotion influence goal attainment in interactions with colleagues and superiors at work.

As will be reviewed in more detail below, research on emotions suggests that emotions and emotion regulation are related to interpersonal consequences in general (e.g., Gross and John, 2003); and to reaching goals specifically (e.g., Scherer et al., 2001); this applies also in the organizational context (e.g., Barsade and Gibson, 2007). On the one hand, *experiencing* positive emotions has been found to foster favorable outcomes in general (e.g., Lyubomirsky et al., 2005) and in the organizational context (for a review, see Ashkanasy, 2003), and to promote proactive goal pursuit in individuals (Bindl et al., 2012). In addition, there also is work on how experiencing emotions by focal persons affects others; the main mechanism by which these effects occur is emotional contagion, which involves a more or less automatic transmission of affective cues to perceivers who, in turn, process, and mimic, these cues more or less automatically as well (e.g., Barger and Grandey, 2006).

Research on *displaying* affect more deliberately comes from two traditions, which are impression management (e.g., Schlenker and Weigold, 1992) and emotional labor (Grandey, 2000). Both support the assumption that expressing positive affect fosters positive social encounters. Among the latter is research on “leading with emotional labor” (e.g., Humphrey et al., 2008; Ashkanasy and Humphrey, 2011b); however, we know much less about how employes try to influence their superiors through affective display, and how that kind of influence compares to effects on peers. Research on emotional labor typically focuses on suppressing emotions one feels and on expressing emotions one does not feel (emotional dissonance, cf. Grandey et al., 2012), but the exaggeration or up-regulation of emotions is often considered part of emotional labor as well (Grandey, 2000).

Up-regulation of positive emotions is arguably especially important for employes low in power, as they are more dependent on creating a positive impression in high-power individuals, who have more means at their disposal to achieve their goals (for instance, they can use negative emotions; Cote et al., 2013). At the same time, exaggerating positive emotion display may increase the danger of appearing inauthentic, which may undermine the intended effects (Liu and Perrewe, 2006). So the question arises whether it may be more effective to just show the positive emotion that is felt, thus delivering a milder, but authentic positive emotion display. We propose that the danger of appearing inauthentic increases to the extent that one has a closer relationship with the interaction partner, which implies that up-regulating positive emotions should be more effective toward supervisors than toward colleagues.

The current study therefore focuses on (a) experiencing and (b) amplifying positive emotions as a means to achieve goals in naturally occurring social interactions at work, assuming that both have different effects on colleagues versus superiors. We focus on the use of positive emotions and their amplification because expressing negative emotions is conducive to goal attainment only in special circumstances (Cote et al., 2013), whereas positive emotions are likely to foster goal attainment almost ubiquitously. The question of authenticity when expressing positive emotions one does not feel has been the focus of quite some research (Hochschild, 1983; Ashforth and Humphrey, 1995; Grandey et al., 2005a). In the context of positive emotions one *does* feel, up-regulating them in one’s display has special implications for the issue of authenticity, in that amplification would seem less necessary if one already feels positive emotions; it therefore may be less effective to up-regulate them in one’s display and thus take the risk of appearing inauthentic.

Our article unfolds as follows: we first discuss how the social functions perspective on emotions can help in explaining the effect of expressing and amplifying positive emotions on goal attainment. We then discuss empirical research concerning the display of positive emotions in relation to goal attainment at work. Finally, we present arguments that such an effect may depend on different interaction partners, specifically, superiors or colleagues.

### Expressing positive emotion and goal attainment in interactions: Mechanisms

With regard to the processes underlying the effect of expressing and managing emotions on goal attainment, we draw on research related to the social functions of emotions, particularly to their informative, influential, and affiliative functions.

First, according to the Emotion as Social Information Model, expression of emotions is a source of information for interaction partners (Van Kleef, 2010; see also Izard, 1977; Ekman, 2003; Cote, 2005). Emotional expression provides information about one’s goals, motivation, and intentions (Van Kleef, 2010, p. 16). Displayed positive emotions signals tendencies to approach a goal (Lyubomirsky et al., 2005), social readiness (Shiota et al., 2004), and the intention to engage in pleasant social interactions (e.g., Keltner and Kring, 1998); these elements are likely to influence an interaction partner to react favorably (Lopes et al., 2005).

Second, expressing emotions is a form of social influence that evokes responses in the interaction partner(s) with regard to attitudes, emotions, thoughts, and behaviors (Kopelman et al., 2008; Niven et al., 2009; Côté and Hideg, 2011). Positive expression conveys a favorable impression (Harker and Keltner, 2001), for instance in terms of friendliness and competence (Barger and Grandey, 2006), which enhances in others the tendency to conform and comply (Cialdini and Goldstein, 2004). Positive expression such as laughter could work as an incentive to induce desirable behavior in others (Staw et al., 1994; Morris and Keltner, 2000).

Furthermore, as mentioned above, expressed emotions influence the *emotions* of others (Zapf, 2002; Niven et al., 2011) via contagion (Hatfield et al., 1994), social appraisal (Zaalberg et al., 2004; Parkinson and Simons, 2009), and social sharing of emotions (Rimé et al., 1998). According to Fredrickson (1998, 2004), positive emotions felt broaden people’s thought-action and behavioral repertoires; these broadened thoughts and behaviors could further promote goal pursuit. Positive mood is also linked to a higher probability of prosocial behaviors (Batson and Powell, 2003; Potworowski and Kopelman, 2008), and it triggers more helping and support (Isen and Simmonds, 1978; George, 1991), more reciprocity (Gouldner, 1960; Walter and Bruch, 2008), more information sharing (Baron et al., 1990, 1992), and also higher tendencies to seek integrative solutions (Forgas, 1998). Barsade (2002) found that the expression of positive emotions by a group member not only might “*ripple out*” among members of the group, it further predicts improved cooperation, decreased conflict, and increased perceived task performance in group setting.

Finally, goal attainment could also be fostered through forming and maintaining good relationships due to the presence of positive emotions in the interactions (Manstead and Fischer, 2000; Shiota et al., 2004). Expressing positive emotions is seen as an affirmation of an agreeable relationship (Fisher and Shapiro, 2006), which enhances social connectedness (Mauss et al., 2011), strengthens group attachment (Lawler, 1992), increases trust (Dunn and Schweitzer, 2005), and improves the emotional climate in groups (Scherer and Tran, 2003). For example, Sy et al. (2005) found that leader’s positive mood could induce positive mood in the team members, and create a positive affective tone in the group. All these effects from positive expression could further foster cooperation (Fischer et al., 2004) and encourage desired behavior in others (Ashkanasy and Humphrey, 2011b); thus, they are likely to foster goal attainment in interactions.

### Expressing positive emotions and goal attainment in interactions: Evidence

Evidence indicating that the expression and amplification of positive emotions could be helpful for attaining goals in interactions at work comes from three sources. First, research on *impression management* explains how people convey a specific, most often a desirable, image of themselves upon others in order to influence outcomes at work (Giacalone and Rosenfeld, 1989; Schlenker and Weigold, 1992). Successful goal pursuit in organizations is influenced by how well people present themselves, interact with and work with others, particularly with their superior and colleagues (Baumeister, 1989). Impression management helps building a positive professional image (Roberts, 2005) and has been found to be related to positive outcomes such as overall career success (Judge and Bretz, 1994), higher salary (Kipnis and Schmidt, 1988), and better performance evaluations (Higgins et al., 2003). Impression management research does not specifically focus on emotions, as employes use various impression management strategies to accomplish goals (Kipnis et al., 1980; Rosenfeld et al., 1995). However, managing emotion expression is one of those strategies (Jones and Pittman, 1982; Grandey et al., 2005a; Andrade and Ho, 2009). Specifically, the two strategies of impression management that have been shown to have the most consistent effects are ingratiation and flattery (e.g., Kipnis and Schmidt, 1988); both imply the expression of positive emotions (Higgins et al., 2003; Harris et al., 2007), and are often used in interactions with superiors (Baumeister, 1989). Second, research *on emotion work* or *emotional labor* (Hochschild, 1979; Zapf and Holz, 2006) has found that the regulation of emotions helps reaching goals during social interactions in organizations, with a particular focus on interactions with clients (Mesmer-Magnus et al., 2012). This line of research shows that displaying positive emotions often leads to favorable outcomes in interactions with clients (e.g., Barger and Grandey, 2006). Expressing positive emotions is associated with more task effectiveness (Rafaeli and Sutton, 1989; Ashforth and Humphrey, 1993), higher customer satisfaction (Pugh, 2001), higher perceived service friendliness, higher chances of customers to return to a store (Tsai, 2001), and better financial outcomes such as higher sales and more tips (Rafaeli and Sutton, 1987). A third tradition indicating that the expression of positive emotions may be helpful in social interactions focuses on emotional contagion (Pugh, 2001; Barsade, 2002; George, 2002; Barger and Grandey, 2006). Research in this area shows that people who experience positive emotions often transmit these emotions to others, which typically has positive effects. However, evidence from this tradition is more indirect, in that its main focus is not on deliberate attempts at transmitting positive emotions.

Together, research on impression management, on emotion work, and on emotional contagion indicate that expressing positive emotions at work may help employes to attain their goals. Furthermore, this research suggests that it is the emotion *expressed*, regardless of the emotion *felt*, that is crucial for the desired effect (Andrade and Ho, 2009), provided that the emotional expression is perceived as authentic and the truly felt emotion does not “leak” through (Grandey et al., 2005a; Liu and Perrewe, 2006; Cote et al., 2013).

With regard to the effect on goal attainment of displaying positive emotions in everyday interactions at work, both impression management research and emotional labor research have some important limitations. The impression management literature describes a very broad array of self-presentation strategies – including appearance, communication content, and behavior (Kipnis et al., 1980); each of them encompasses much more than the display of emotions. The display of positive emotions is implied in some of the tactics described, but often it is not specifically investigated. Concerning emotion work, the majority of studies emphasize how the display and the regulation of emotions influence *intra*personal outcomes, such as individual well-being (Giardini and Frese, 2006), job satisfaction (Pugliesi, 1999; Grandey et al., 2005b), and stress (Zapf et al., 2001; Brotheridge and Grandey, 2002; Grandey, 2003; Totterdell and Holman, 2003; Grandey et al., 2005b). There are results that refer to interactional goals (e.g., getting more tips; Rafaeli and Sutton, 1987; see above), but these typically refer to strangers (clients, customers, etc.). In interactions with people that one interacts with on a daily basis, such as colleagues and superiors, these strategies may not be as effective (e.g., because these interaction partners are more skilled in detecting them, or because authenticity may be a strong norm); however, with few exceptions (Tschan et al., 2005), superiors and colleagues as interaction partners have not been in the focus of emotion work research. Furthermore, when dealing with emotion displays that are not in accordance with one’s feelings (i.e., surface acting), emotional labor research typically focuses on the suppression of negative emotion and their masking by either neutral or positive emotion display. The up-regulation of positive emotions that one does feel has not received much attention (see Nair, 2008; Cote et al., 2013), nor has the fact that in such a case it may suffice to express the emotion felt, thus showing a weaker expression but avoiding the danger of perceived inauthenticity.

In sum, research on impression management and emotion work provides much general evidence that managing the expression of emotions in interaction is likely to be related to goal attainment, but they are not very specific with regard to expressing emotions (impression management) or they focus on strangers rather than people one interacts with frequently at work, and on the display of positive emotions that are not felt (emotional labor).

### Emotion display and interaction partners: Superior versus colleagues

Strategic emotion expression or the display regulation of emotion strongly depends on the type of interaction partner (Clark et al., 1996). To reach goals, people are likely to selectively focus their emotion regulation behavior toward more important interaction partners, especially those who have power and control over their outcomes in organizations (Kilduff et al., 2010). At the same time, it is also plausible that the *effect* of emotional expression, and particularly the effect of display regulation, on goal attainment depend on the interaction partner. Specifically, we assume that expressing, and amplifying, positive emotions should have a greater impact in interactions with superiors as compared to colleagues. Two aspects of the relationships involved are especially important for our reasoning: familiarity (closeness), and hierarchy (power) (e.g., Zaalberg et al., 2004; Clark and Finkel, 2005; Hall et al., 2007; Glaso and Einarsen, 2008).

First, more frequent, and more informal, interactions between colleagues (as compared to interactions with supervisors) imply higher familiarity (cf. Argyle and Henderson, 1985; Kahn, 2007), which, in turn, implies that one knows the other person comparatively well and may evaluate his or her behavior more in terms of its contribution to the common work goal (e.g., dependability, cooperativeness, supportive behavior, etc.) than in terms of the way the behavior is expressed. In other words, colleagues may be willing to comply with a request even if it is not accompanied by the expression of positive emotions. Such compliance would be in line with the “*rules for coworkers*” investigated by Argyle and Henderson (1985), according to which colleagues are expected to cooperate on common goals independent of the quality of their relationship. The evidence on actual behaviors in the workplace is line with this reasoning. Thus, people perform less emotion work with interaction partners who are closer to themselves as compared to more distant interaction partners (Diefendorff et al., 2010). A recent event-sampling study found that people engage in more effortful impression management with distant than with close others (Gosnell et al., 2011). In closer relationships, other considerations, especially authenticity, seem to gain more weight. Most employes have closer relationships among each other than with their superiors (Argyle and Henderson, 1985). In closer relationships, faking unfelt emotions is generally not well-received; individuals are expected to interact more authentically, openly, and honestly (Clark et al., 1996). People do, indeed, express their emotions more authentically to their coworkers than to their superior (Diefendorff et al., 2010). Colleagues are more likely than strangers to detect an inauthentic positive emotion display, causing this tactic to “backfire,” and potentially ruining one’s credibility and one’s reputation (Clark et al., 1996). (Such backfiring effects are not confined to colleagues; they have been reported for more distant interaction partners, such as clients (Grandey et al., 2005a). However, as employes usually are in closer contact with their colleagues than with their superiors, the chance of “being caught” is likely to be higher in interactions with colleagues). Therefore, expressing and amplifying positive emotions may be less effective in a relationship that is high in familiarity. In contrast, a superior with whom one has a more distant relationship is less likely to detect (at least subtle) signs of emotion regulation; he or she might rely more strongly on the emotional expression projected by a subordinate when judging the subordinate’s emotion (Ashkanasy and Humphrey, 2011b, p. 37); as discussed previously, showing positive emotions toward a superior would be advantageous from this perspective.

Second, being hierarchically lower than the interaction partner, and therefore having less power, implies that one depends on the goodwill of the interaction partner to a much greater extent than when one deals with colleagues of equal standing. Among colleagues, work goals are often imposed on everyone by the organization, and thus, cooperation toward goals in interactions is less discretionary. This lack of discretion is also implied by the fact that colleagues often depend more strongly on each other, which makes reciprocity especially salient and entails greater risks for a tit-for-tat response of a colleague whose interests have been ignored. In contrast, supervisors have more discretion with regard to going along with requests by subordinates or for supporting their specific goals. This power position allows them to be influenced more strongly by momentary signs of cooperativeness and compliance by the subordinate, and to react more strongly to their own mood when making a decision. It also is possible that they are easily flattered, attributing positive emotion display to their convincing and “winning” way of interacting and leading (cf. Pfeffer et al., 1998), thus becoming victims of the “romance of leadership” themselves (Gray and Densten, 2007). Since one of the important aspects of expressing positive emotions is that it may induce a positive mood in others (Hatfield et al., 1994; Zaalberg et al., 2004; Parkinson and Simons, 2009; Niven et al., 2011), these aspects are likely to play a greater role for superiors as compared to colleagues.

Research on actual behavior toward supervisors is in line with our reasoning. For instance, Mann (1999) showed that low status individuals engaged in more display regulation than high status individuals, and research by Méhu (2011) showed that people use more strategic smiles when interacting with people of higher status. In a similar vein, flight attendants expressed more positive emotions toward first and business class passengers than to economy class passengers (Hochschild, 1983). In organizations, employes engaged in less emotion work when dealing with partners of equal or lower status (colleagues) as compared to clients (Tschan et al., 2005) or superiors (Diefendorff et al., 2010). Also, impression management tactics frequently involve *upward* influence tactics (Kipnis and Schmidt, 1988), and employes express positive emotions to foster positive outcomes at work (Wayne and Liden, 1995). Research on impression management shows that people adapt their tactics to the perceived power of the audience (Gardner and Martinko, 1988) and its expectations (Rudman, 1998), and that they use specific impression-management tactics in interactions with superiors (Baumeister, 1989). It seems likely that subordinates are especially vigilant toward their superiors and monitor closely how the superiors react to their behaviors, thus putting special effort into adjusting their behaviors, including their emotion display, to the signals of receptivity sent by the superiors (Kilduff et al., 2010). Furthermore, Staw et al. (1994) found an effect of positive emotions on social support from both colleagues and supervisors; however, this effect was stronger for support by superiors as compared to colleagues. Thus, showing positive emotions seems to be more important, and more effective, when dealing with superiors, as opposed to colleagues, and actual behavior is in accordance with this assumption. Note that we are talking about the likelihood of reacting in a specific way in specific situations; thus, when we say that superiors may let themselves be guided by their mood more than subordinates, we do not imply that they do this consistently. For instance, it seems likely that employes adjust their emotion display to situational characteristics that signal favorability for pursuing their goals (Kilduff et al., 2010).

### Current study

The aim of the present research is to investigate if the expression of positive emotions and the enhancement of positive emotions (i.e., amplifying the display of positive emotions felt) facilitate achieving goals during naturally occurring social interactions at work. We examine this issue (a) in general, and (b) with regard to different interaction partners, specifically colleagues and superiors.

We state our hypotheses as follows:

Hypothesis 1. A stronger expression of positive emotions during interactions at work will be related to a higher level of goal attainment.

Given that a positive emotion expression could be due to the actual positive emotion felt, its expression may be based on two processes. First, the intensity of the emotion display may correspond to the intensity of the emotion felt; second, it may be based on display regulation involving its amplification in comparison to the intensity it is felt (cf. Gross, 1998). We emphasized above that it is the expression of positive emotions that is responsible for positive effects in social interactions, not the underlying emotion itself, at least as long as the emotion display is perceived as authentic by the interaction partner, which may often be the case. Amplifying a positive emotion, that is, displaying it with a higher intensity than it is felt, may, therefore, represent a promising strategy for achieving goals. These considerations lead to the following hypothesis:

Hypothesis 2. Employes’ *amplification of positive emotions* during a workplace interaction is related to a higher level of goal attainment during the interaction.

Based on the arguments presented above, we also posit that the type of interaction partner (superior versus colleague) moderates the relationship between expressing, as well as amplifying, positive emotions and the degree of goal attainment in everyday interactions at work. More specifically, we suppose that expressing as well as amplifying positive emotions has a stronger relationship to goal attainment during interactions with superiors than during interactions with colleagues.

Hypothesis 3. The interaction partner moderates the relationship between the expression of positive emotions and goal attainment in the sense that this relationship is stronger for interactions with superiors than for interactions with colleagues.

A similar assumption is formulated for amplifying positive emotions.

Hypothesis 4. The interaction partner moderates the relationship between amplifying positive emotions and goal attainment in the sense that this relationship is stronger for interactions with superiors than for interactions with colleagues.

## Materials and Methods

### Participants

We recruited 113 Swiss employes from different organizations, using a snow ball recruiting system. Of the participants, 61.75% were women, mean age was 34.3 years (SD = 13.8), age ranged from 18 to 66. Level of education ranged from basic training to the completion of a professional or tertiary degree; participants worked in a wide range of occupations across different sectors of employment. Participation was voluntary and not compensated.

### Study design and procedure

We conducted the study using a variant of the Rochester Interaction Record methodology (Reis and Wheeler, 1991) to sample everyday interactions at work. Participants were first asked to complete a general questionnaire containing demographic questions, a personality scale, and job-related questions. They were then asked to record each interaction they had over a 7-day period, and to answer questions about each interaction. Before the self-observation period, participants met with a research assistant who handed them the general questionnaire and seven daily booklets for recording the interactions. They were instructed on how to use the interaction records. We asked them to answer the questions as soon as possible after every social interaction that lasted 10 min or longer, and on shorter interactions they considered important. They were informed that this study was about investigating emotions in daily life during social interactions at work and in private life. The research assistant explained what we meant by an interaction (an encounter with one or more other people during which they mutually adjusted their behavior); and what was not considered an interaction (e.g., waiting for a bus with other people). Together with the research assistants, participants filled out sample interaction records to familiarize themselves with the methodology. Participants filled in the general questionnaire the same day and started the 7-day interaction record period the next day. They reported interactions for each day in separate daily booklets and mailed the booklets back to the researchers. The study was conducted in French; all non-French-language instruments were translated into French and controlled by back-translation.

### Measures

#### General questionnaire (measures on the person-level)

We recorded participants’ demographics such as sex, age, level of education, occupation, and the nature of their jobs. We measured neuroticism and extraversion by administrating the Big Five Personality Test (Costa and McCrae, 1995), in a short version developed by Schallberger and Venetz (1999). Cronbach’s alpha for neuroticism and extraversion was 0.77 and 0.74, respectively.

#### Daily interaction records (measures on the interaction-level)

For each interaction, participants indicated whether it took place at work or outside of work. Only interactions at work were considered for this study. For each interaction, participants answered several questions, including whether they pursued a goal during the interaction. Only interactions for which goal pursuit was reported were included in the study.

##### Interaction partners

Participants provided information about the type of interaction partners for each interaction (colleague, superior, client, other). As the focus of this study is on interactions with superiors and colleagues, we excluded interactions involving only clients or other interaction partners. We created a dummy variable representing the presence of the superior in the interaction (0 = only colleagues are present; 1 = superior is present).

##### Emotions experienced and emotions shown during the interactions

For each interaction, participants were asked to report the emotion(s) felt and the emotion(s) shown during the interactions, using a variant of the Geneva Emotion Wheel (Scherer, 2005). The Geneva Emotion Wheel is a graphical tool that allows participants to record discrete positive emotions (e.g., interest, joy, pride etc.) and discrete negative emotions (e.g., anger, disappointment, shame etc.) as well as the *intensity* of each emotion on a scale from 1 to 4 on circles with increasing size, with an option to indicate “none” in the middle of the wheel. If an emotion was not ticked, it was coded as 0 (not felt or not shown, respectively). The Geneva Emotion Wheel is an accessible, easy to use tool that has been successfully used under time pressure and for repeated assessments (Tran, 2004; Hunziker et al., 2011; Scherer et al., in press). Two sets of the Geneva Emotion Wheel were used for each interaction, referring (1) to emotions experienced and (2) to emotions shown. *Emotions experienced* were measured on the first emotion wheel by asking “*In this interaction, which emotion(s) did you feel?* *Indicate all emotions felt as well as their intensity on the emotion wheel*.” *Emotions shown* were measured on the second emotion wheel by asking “*In this interaction, which emotion(s) did you show? Indicate all emotions you showed as well as their intensity on the emotion wheel*.” We computed scores for positive emotions by calculating the mean intensity of the emotions interest, happiness, joy, pleasure, tenderness, enthusiasm, relief, and compassion for emotions felt as well as for emotions shown. We computed scores for negative emotions shown and felt as the mean intensity of anger, contempt, disgust, disappointment, anxiety, sadness, embarrassment, shame, and guilt in an analogous way.

##### Degree of goal attainment during the interaction

To measure the degree of goal attainment in the interaction, participant answered the question “*Have you attained your objective(s) in this interaction?*” on a five point Likert scale from 1 (not at all) to 5 (absolutely).

#### Analyses

As interactions are nested within individuals, we analyzed the data by way of multilevel regression analysis (Nezlek, 2003; Hox, 2010) using SPSS (Heck et al., 2010). Interactions are represented on level 1 (interaction-level), and individual participants are represented on level 2 (person-level).

For Hypotheses 1 and 3, which refer to the expression of positive emotions, we used multilevel regression analysis. For testing Hypotheses 2 and 4, which refer to the enhancement of positive emotion (i.e., the discrepancy between positive emotion felt and positive emotion shown), we ran polynomial procedures as suggested by Shanock et al. (2010). Following Hu and Liden (2012) and Vidyarthi et al. (2010), who ran polynomial analyses within a multilevel structure, we included the higher level terms of positive emotion felt and positive emotion shown; however, if the test of the curvature of the estimated response surface, which consists of the higher level terms (i.e., Felt^2^ − Felt × Shown + Shown^2^), was not significant, we proceeded with the linear terms only and computed the discrepancies of positive emotion by subtracting the regression coefficient of positive shown from the regression coefficient for positive felt (see Vidyarthi et al., 2010; Hu and Liden, 2012). Finally, we tested the slope of incongruence by surface response tests (Shanock et al., 2010).

For all of our analyses, we included control variables that have been found to covariate with emotional constructs in social contexts. We controlled for age, as it has been shown that a shift in emotion regulation strategies is associated with developmental changes in adulthood (John and Gross, 2004). We controlled for gender, as there are gender differences in participation in social interactions (Wheeler and Nezlek, 1977) and in emotional suppression (Gross and John, 2003). We controlled for extraversion and neuroticism as these personality traits have been found to influence individual’s susceptibility for experiencing emotions (Watson et al., 1988; Brotheridge and Grandey, 2002; Diefendorff and Richard, 2003; Diefendorff et al., 2011). Neuroticism and extraversion are related to higher emotional expressivity (Gross and John, 1994), and extraversion is related to display regulation (Diefendorff et al., 2005; Judge et al., 2009). At the interaction-level, we controlled for positive and negative emotion experienced or emotion shown whenever appropriate.

In terms of centering, for all person-level variables where zero was not a meaningful number, we used grand mean centering (GMC). For all continuous interaction-level variables, we chose a centering method that corresponded with our method of analysis. In multilevel analysis (Hypotheses 1 and 3) we used group mean centering (CWC), as suggested for this type of research (Enders and Tofighi, 2007; Hox, 2010; Ohly et al., 2010). For polynomial regression (Hypotheses 2 and 4), we used GMC (Edwards and Parry, 1993).

## Results

### Descriptive statistics

Participants reported a total of 1535 interactions at work, corresponding to a mean of 13.58 interactions per participant. Of those interactions, 930 were with superiors and/or with colleagues. Participants reported pursuing a goal in 72.9% of the interactions with the superior present, and in 47.3% of the interactions with colleagues present. In total, 494 interactions were included in the analyses, which all involved interactions with superiors and/or colleagues as well as goal pursuit. Mean goal attainment per interaction was 3.93 (SD = 1.19).

Table 1 shows the means, standard deviations, and intercorrelations of all person-level variables; Table 2 shows the means, standard deviations, and intercorrelations of the interaction-level variables.

**Table 1:** **Means, standard deviations, and correlations between level 2 variables**.

	Range	*M*	SD	1	2	3
Gender	Female = 0, Male = 1	0.38	0.49	1		
Age	18–66	35.26	14.28	−0.11	1	
Extraversion	1–6	4.19	0.83	−0.02	−0.21*	1
Neuroticism	1–6	2.80	0.80	−0.22*	0.12	−0.23*

*N = 112 employes*.

^†^p < 0.10, *p < 0.05, **p < 0.01 (two-tailed).

**Table 2:** **Means, standard deviations, and correlations between level 1 variables**.

	Range	*M*	SD	1	2	3	4	5	6
Positive emotion felt	0–4	0.78	0.65	1				
Positive emotion expressed	0–4	0.75	0.58	0.84**	1			
Negative emotion felt	0–4	0.24	0.37	−0.13**	−0.13**	1		
Negative emotion expressed	0–4	0.11	0.29	−0.17**	−0.19**	0.72**	1	
Amplification of positive emotion	0–4	0.13	0.24	−0.04	0.40**	0.08	−0.11*	1
Superior present (yes = 1; no = 0)	0, 1	0.34	0.47	−0.11*	−0.08^†^	0.04	0.05	−0.04	1
Degree of goal attainment	1–5	3.9	1.2	0.32**	0.29**	−0.41**	−0.30**	0.04	−0.02

*n = 494 interactions at work with goal pursuit with superiors and/or with colleagues*.

^†^p < 0.10, *p < 0.05, **p < 0.01 (two-tailed).

### Positive emotions expressed and goal attainment

The initial analysis of an unconditional null model without any predictors confirmed that it was appropriate to use multilevel analysis. The intercept varied significantly across individuals (Wald *Z* = 2.958, *p* < 0.001), and the intraclass correlation (ICC) of 0.17 suggested that a large amount of the variability in the degree of goal attainment resided within individuals (Heck et al., 2010).

Hypothesis 1 states that positive emotions expressed during the interaction (whether from genuine emotions felt or from amplification) are related to goal attainment; Hypothesis 3 states that this relationship is moderated by interaction partner in that the relationship between positive emotions expressed and goal attainment is stronger in interactions with superiors than in interactions with colleagues.

Results are displayed in Table 3. To test Hypotheses 1 and 3, we first estimated a two-level unconditional null model. Model 1 in Table 3 shows the results for Hypothesis 1. Besides our predictor variable positive emotions expressed we included the control variables age, gender, extraversion, and neuroticism on the person-level, and negative emotions expressed on the interaction-level. Expression of positive emotions during the interaction was significantly related to the degree of goal attainment (*B* = 0.80, SE = 0.13, *p* < 0.01), supporting Hypothesis 1. Note that the expression of negative emotions also showed a (negative) relationship to goal attainment (*B* = −0.54, SE = 0.23; *p* < 0.05). Of the control variables, only neuroticism was marginally related to goal attainment.

**Table 3:** **Predicting goal attainment in workplace interactions by expressing positive emotions (Hypothesis 1 and Hypothesis 3)**.

Variables	Unconditional	Model 1 (Hypothesis 1)	Model 2 (Hypothesis 3)
	Estimate (SE)	Estimate (SE)	Estimate (SE)
Intercept	3.91 (0.07)**	3.97 (0.10)**	3.99 (0.10)**
**LEVEL 2 (GRAND MEAN CENTERED)**			
Gender (female = 0, male = 1)		−0.12 (0.15)	−0.13 (0.15)
Age		0.00 (0.01)	0.00 (0.01)
Extraversion		0.01 (0.09)	0.03 (0.09)
Neuroticism		−0.18 (0.09)^†^	−0.18 (0.09)^†^
**LEVEL 1 (GROUP MEAN CENTERED)**			
Positive emotions expressed		0.80 (0.13)**	0.59 (0.15)**
Negative emotions expressed		−0.54 (0.23)*	−0.50 (0.23)*
Superior present (yes = 1; no = 0)			−0.02 (0.11)
Interaction term:			
Positive shown × superior present			0.72 (0.28)**

*N = 113 employes, n = 494 interactions at work involving goal pursuit with superiors and/or with colleagues*.

^†^p < 0.10, *p < 0.05, **p < 0.01. Tests are all two-tailed.

Hypothesis 3 postulated a moderating effect of the interaction partner. It was tested by adding the interaction partner variable (superior present versus only colleague(s) present), and subsequently the interaction term of positive expression times interaction partner to the previous model. The interaction term was significant (*B* = 0.72; SE = 0.28, *p* < 0.01).

**Figure 1:**
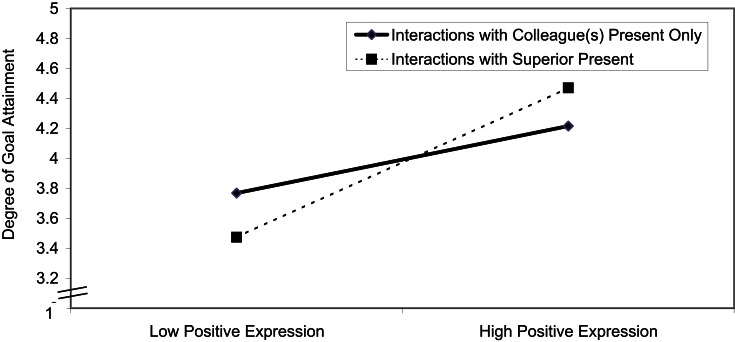
**Predicting goal attainment by *expressing* positive emotions during interactions at work with a superior present versus not present**.

To illustrate the direction of the effect, we present the result in Figure 1 as an interaction plot (Dawson and Richter, 2006), containing separate regression lines for interactions with colleagues and for interactions with superiors. Figure 1 indicates that expressing positive emotions was more strongly related to goal attainment in interactions with superiors, as compared to interactions with colleagues. A single slope test (Preacher et al., 2006) showed that the slope for interactions with superiors was significantly different from zero (*t* = 2.57, *p* = 0.01), whereas the slope for interactions with colleagues was not (*t* = 1.23, *p* = 0.23). These results support Hypothesis 3.

### Amplifying the expression of positive emotions and goal attainment

In Hypothesis 2 we state that the amplification of positive emotions felt (i.e., showing positive emotions more strongly than they are felt) is related to higher goal attainment in work-related interactions; Hypothesis 4 states that this relationship is more pronounced in interactions with superiors than in interactions with colleagues.

Results are presented in Table 4. Again, age, gender, extraversion, and neuroticism were included as control variables on the person-level. In these analyses, we entered both positive felt and positive shown emotions, which allows for assessing the effect of congruence between positive felt and shown (i.e., authentic positive emotion expression), and the effect of incongruence between positive felt and shown (i.e., the enhancement of positive, and the suppression of positive emotion). In the analyses of emotion display (Table 3), expressing negative emotions was significantly associated with lower goal attainment. For the analysis of amplification effects (Table 4), we also controlled for negative emotions, both felt and shown. Indeed, negative emotions felt were significantly associated with low goal attainment, both overall and in the analyses involving superiors or colleagues, respectively. Following Hu and Liden (2012), the higher level terms for positive emotion (i.e., Felt^2^ − Felt × Shown + Shown^2^) were not included in the final model, as they were insignificant in all analyses, indicating the absence of non-linear relationships (see the section on analyses).

**Table 4:** **Predicting goal attainment in workplace interactions from positive felt and shown (Hypothesis 2 and Hypothesis 4)**.

Variables	All partners (Hypothesis 2)	Superior (Hypothesis 4)	Coworker (Hypothesis 4)
	Estimate(SE)	Estimate(SE)	Estimate(SE)
Intercept	4.15 (0.08)**	4.32 (0.13)**	4.14 (0.09)**
**LEVEL 2 (GRAND MEAN CENTERED)**			
Gender (female = 0, male = 1)	−0.11 (0.12)	−0.48 (0.18)*	−0.07 (0.13)
Age	0.00 (0.00)	−0.02 (0.01)*	0.00 (0.00)
Extraversion	0.02 (0.07)	−0.11 (11)	0.04 (0.08)
Neuroticism	−0.06 (0.08)	−0.11 (0.12)	−0.05 (0.08)
**LEVEL 1 (GRAND MEAN CENTERED)**			
Positive felt	0.37 (0.14)*	−0.12 (0.26)	0.44 (0.15)**
Positive shown	0.17 (0.15)	0.93 (0.29)**	−0.05 (0.17)
Congruence between positive felt and shown	0.51 (0.09)**	0.81 (0.16)**	0.39 (0.10)**
Discrepancy between positive felt and shown	**0.16 (0.28)**	−1.05 (0.53)*	**0.50 (0.31)**
Control variables			
Negative felt	−1.31 (0.18)**	−1.24 (0.26)**	−1.38 (0.22)**
Negative shown	0.43 (0.25)^†^	0.35 (0.34)	0.66 (0.35)^†^

*N = 113 employes, n = 494 interactions at work involving goal pursuit with superiors and/or with colleagues*.

^†^p < 0.10, *p < 0.05, **p < 0.01. Tests are all two-tailed.

**Figure 2:**
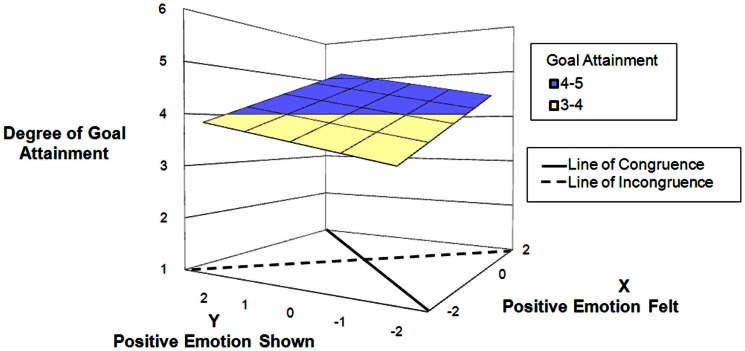
**Predicting goal attainment by positive emotions felt and shown during interactions at work**.

Hypothesis 2 postulated an effect of amplifying positive emotions regardless of the interaction partner. The response surface slope test for the line of congruence (*x* = *y*) was highly significant (*B* = 0.51, SE = 0.09, *p* = 0.001), suggesting that there is a positive linear relationship between authentic positive expression and degree of goal attainment. However, the response surface slope test for the line of incongruence (*x* = −*y*) was not significant, suggesting that neither enhancement nor suppression of positive emotion influenced degree of goal attainment. Amplification of positive emotions therefore does not seem to enhance goal attainment in general; Hypothesis 2 is thus not supported. These results are displayed in Figure 2.

**Figure 3:**
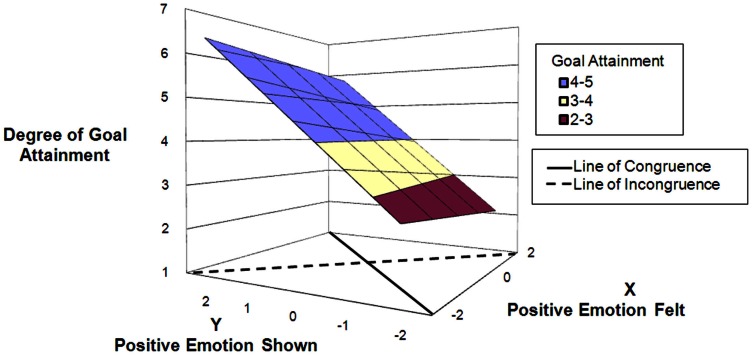
**Predicting goal attainment by positive emotions felt and shown during interactions with *superiors***.

**Figure 4:**
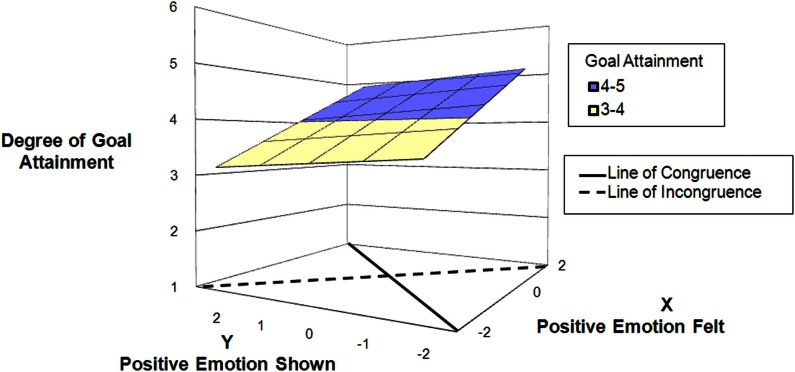
**Predicting goal attainment by positive emotions felt and shown during interactions with *coworkers***.

Hypothesis 4 postulated that the effect of amplifying positive emotions would be stronger for superiors as compared to colleagues as interaction partners. To assess differences between interaction partners, we ran separate analyses for interactions with superior present, and for interactions with colleague(s) present. Results support Hypothesis 4 (Table 4, Model 4). For encounters with a superior (displayed in Figure 3), the response surface slope test for the line of congruence (*x* = *y*) was highly significant. (*B* = 0.81, SE = 0.16, *p* = 0.001) suggesting that there is a positive linear relationship between authentic positive expression and degree of goal attainment. Most importantly, the response surface slope test for the line of incongruence (*x* = −*y*) was significant. (*B* = −1.05, SE = 0.53, *p* = 0.047 two-tailed). The negative sign of the coefficients implies the effect on goal attainment is driven by showing more positive emotions than felt; thus it is the enhancement of positive shown, not the suppression of positive emotion that is important for achieving goals. For encounters with colleagues (displayed in Figure 4), the response surface slope test for the line of congruence (*x* = *y*) was highly significant. (*B* = 0.39, SE = 0.10, *p* = 0.001) suggesting that there is a positive linear relationship between authentic positive expression and degree of goal attainment. The response surface slope test for the line of incongruence (*x* = −*y*) was not significant, suggesting that neither enhancement nor suppression of positive emotions influence degree of goal attainment. These results support Hypothesis 4.

### Alternative analysis

With regard to Hypotheses 2 and 4, we considered several ways of conducting these analyses besides multilevel polynomial analysis. One involves an interaction between emotion felt and emotion shown, and the other involves the creation of an emotion enhancement score (i.e., a difference score). All these analyses led essentially to the same results; the interaction plot (Dawson and Richter, 2006) for enhancing positive expression is similar to Figure 1; the slope test (Preacher et al., 2006) showed that more amplification of positive emotions was related to higher levels of goal attainment only in interactions with superiors (*t* = 2.48, *p* = 0.01), but not in interactions with colleagues (*t* = 0.28, *p* = 0.78).

## Discussion

We investigated the effects of expressing and amplifying the expression of positive emotions in interactions with colleagues and/or superiors at work on goal attainment. In more than half (53.1%) of the interactions participants reported having pursued a goal; this underscores the importance of goals in interactions at work. Although the degree of goal attainment was relatively high (AM = 3.9 on a scale from one to five), we did find relationships between emotions expressed and goal attainment and between display regulation and goal attainment. We were interested in whether expressing and amplifying *positive* emotions is related to the degree of goal attainment in social interactions at work. The results, based on 494 interactions at work provided by 113 employes, suggest that (1) the *expression* of positive emotions is related to higher goal attainment, but (2) this main effect is qualified by an interaction indicating that this effect only holds for interactions with superiors, not for interactions with colleagues. The results furthermore (3) suggest that *amplifying positive emotions* in interactions is significantly related to goal attainment in interactions with superiors, but not in interactions with colleagues.

We discuss (1) the expression of positive emotions and the role of authenticity in general, and (2) the differential findings for interactions with coworkers and superiors.

(1) Our result of a significant main effect of expressing positive emotions is in accordance with previous research that tested similar effects in a more indirect way or by experimental research. For example, negotiation research has shown that people in a positive mood are more likely to adopt optimistic, cooperative strategies, and seek integrative solutions (e.g., Carnevale and Isen, 1986; Forgas, 1998), and less likely to engage in aggressive tactics (e.g., Baron, 1984), thus contributing to better joint outcomes (Potworowski and Kopelman, 2008). Our findings are also in accordance with the literature on social functions of emotions (Clark et al., 1996; Van Kleef, 2010), which suggests that expressing positive emotions may be perceived by the interaction partner as signaling cooperation, which could be functional for goal attainment.

Note that effects of expressing positive emotions cannot be attributed to an absence of negative emotions, as expressing negative emotions were controlled for in our analyses. Not unexpectedly (although not hypothesized, as it was not the focus of this paper), we found a negative effect of expressing negative emotions on goal achievement. Again, this is in accordance with previous studies. For example, Friedman et al. (2004) showed that in real electronic mediations, expressing anger reduced settlement quality. Our finding that expressing negative emotions is negatively related to reaching goals thus replicates these earlier findings. Note that expressing anger has been found to predict better outcomes for the person expressing anger in some specific circumstances, such as short term negotiations among strangers (Van Kleef et al., 2004).

However, our study extends previous research by showing that expressing positive emotions is not conducive for goal attainment unconditionally. Specifically, the effect for expressing positive emotions was moderated by the type of interaction partner: expressing positive emotions increased goal attainment only during interactions with superiors as when compared to during interactions with colleagues; we will comment on that result below.

The polynomial regression analysis offers additional insights. The results of this analysis suggests that expressing positive emotions *authentically* is beneficial regardless of the interaction partner, as the slope for the line of congruence is significant in all three analyses.

It is not surprising that expressing positive emotions authentically has positive effects regardless of the interaction partner. Authentic expression of positive emotions has all the advantages associated with expressing positive emotions that have been postulated, and found, in research on emotional contagion (e.g., Barsade, 2002) and on emotional labor (regarding deep acting and genuine emotional displays; Ashkanasy and Humphrey, 2011a), but it does not contain the risk of “leaking” associated with faking (Grandey et al., 2005a; Liu and Perrewe, 2006).

That the effect of authentic display of positive emotions is not likely to be disputed actually provides the basis for our focus on the way people express positive emotions they actually feel. Most notably, since an authentic expression of these emotions promises positive effects without risks, can one expect any additional effect of amplifying these positive emotions? Amplifying positive emotions might not only yield little additional value, as the underlying emotion felt already is positive; it might actually backfire if it is detected as non-authentic. Thus, there is an important contrast to the issue of negative emotion display. Expressing negative emotions may have such damaging effects that the risk of being detected may seem worth taking in many situations. For positive emotions, the benefits of amplifying them are not so obvious. Showing that amplifying positive emotions may support goal attainment therefore adds to the literature.

We postulated a main effect of amplifying positive emotions on goal attainment in everyday social interactions at work. To formulate our hypotheses we drew, among others, on the impression management literature (Giacalone and Rosenfeld, 1989). Impression management tactics that include expressing and amplifying positive emotions have been found to have the most consistent effects on long-term organizational outcomes (Higgins et al., 2003; Harris et al., 2007). While we did not find an effect for the amplified expression of positive emotions for colleagues as interaction partners, we did find it for supervisors; it is that effect that we turn to now.

(2) We hypothesized that the influence of expressing or amplifying positive emotions on goal attainment is more pronounced in interactions with superiors than in interactions with colleagues, based on considerations concerning power (Mast and Hall, 2004), relationship closeness (Clark and Finkel, 2005), and rules of cooperation at work (Henderson and Argyle, 1986). Multilevel moderated regression analyses supported these contentions, and slope tests revealed that an effect of expressing positive emotions was only found in interactions with superiors, but not in interactions involving colleagues only, as hypothesized. Furthermore, in the polynomial regression analysis, amplifying positive emotions increased goal attainment only in interactions with superiors, but not in interactions with colleagues. These findings are in accordance with research showing that people adapt their tactics to the perceived power of the audience (Gardner and Martinko, 1988) and specifically to situations that involve interacting with superiors (Baumeister, 1989). We argued that this tendency to engage in more emotion regulation vis-a-vis superiors is not only more frequent but also especially effective (cf. the study by Staw et al. (1994), who did not, however, distinguish between emotions felt and shown, and did not refer to daily interactions).

Bound by work rules and norms (Argyle and Henderson, 1985), colleagues typically are dependent on the focal person to a much greater degree than supervisors, which implies that they have less discretion concerning whether or not they will comply with the focal person’s goals; they therefore should be less strongly influenced by the expression of positive emotions than supervisors. Also, for colleagues, the focal person’s behavior is embedded in a much wider and richer context, such as their more intimate knowledge about the dependability, cooperativeness, and contributions of the focal person in general; such a rich context-knowledge should render specific behavioral instances less important for colleagues, as compared to superiors, who often do not have such a rich contextual background knowledge. Furthermore, the chances that faking emotions may backfire should be greater when interacting with colleagues, as they are more likely to detect an inauthentic positive emotion expressed.

In contrast to colleagues, superiors often know the employe less well and therefore may be less likely to detect subtle signs of inauthenticity. Unless there is a specific reason to be very attentive (e.g., when they depend on the cooperation of a specific employe in a given situation; cf. Kilduff et al., 2010), they may not search for pertinent information deeply enough, being satisfied with external signs of positivity. Such a lack of vigilance may be supported by the fact that deliberate smiles are more common in people who are low in status (Méhu, 2011); superiors therefore may simply be used to that kind of behavior and assume it to be normal. One might even speculate that some supervisors may notice the inauthenticity but not be bothered by it; rather, they may interpret such behavior as appropriate for subordinates to display toward their superiors, as they indicate the awareness, and acceptance, of the power differential by the less powerful partner (cf. Méhu and Dunbar, 2008).

All in all, in terms of achieving one’s goals, it seems to pay off to express positive emotions when interacting with superiors, and to even amplify positive emotions that are not strongly felt. There is a certain irony in these findings: Employes tend to *show* *more* positive emotions when superiors are present, as indicated by the positive correlation between the presence of a superior and the expression of positive emotions in Table 2. However, they *experience* *fewer* positive emotions when interacting with superiors as compared to colleagues, as indicated by the negative correlation between the presence of a superior and the experience of positive emotions (see Table 2, and cf. the finding by Tschan et al. (2010) that people experience less pleasure when superiors are present). Emotional labor toward superiors, which so far has been overshadowed by the dominant focus on clients (for an exception, see Tschan et al., 2005), deserves much more attention, as does the question of by which mechanisms employes manage to induce their superiors to comply with their objectives by showing positive emotions.

### Limitations and strength

This study has several limitations. First, all data are based on self-report, which bears the risk of common method bias. There are still limited alternatives to self-report when assessing emotions (De Gelder, 2010), particularly in everyday situations. As self-report bias has been found to be influenced by positive and negative trait affectivity (Podsakoff et al., 2003), we controlled for trait extraversion and trait neuroticism in this research, thus alleviating the common method problem. Note also that we asked questions about feeling and showing emotions and goal attainment in interactions repeatedly; our results could therefore be attributed to common method bias only to the extent that this bias is differentially associated with specific interactions. Also, emotions (felt and shown) and goal attainment are assessed by different types of scales, which also might alleviate the common method problem (Ashkanasy et al., 2006). Finally, common method bias makes it, if anything, more difficult to detect statistical interactions. Note also that a number of authors recently have concluded that the common method problem may have been overstated (e.g., Spector, 2006). Common methods bias may have influenced our results, but it is unlikely that this bias would render the results spurious.

Second, the most important limitation of the study is that we cannot reliably establish cause-effect relations. Information about the interactions, the interaction partners, emotions expressed and the amplification of positive emotions were all measured immediately after the interaction. It is plausible that part of the emotional aspects reported is a result of the degree of goal attainment rather than a predictor of goal attainment. This concern would not be alleviated much by a temporal separation of the measures, as in real life interactions it may become clear already during the interaction whether a goal can be reached or not, and emotional experiences may thus be influenced by this. This concern is particularly important for the interpretation of our results regarding emotions expressed (Hypotheses 1 and 3), because they correlate highly with the emotions felt. However, we feel that the argument applies less for amplification of positive emotions; they were measured as the discrepancy between positive emotions expressed and positive emotions felt, and, in addition, positive emotions felt were controlled for in our polynomial regression analyses. Whereas failure or successful goal attainment are affective events and influence emotions *felt* (Weiss and Cropanzano, 1996), it is theoretically less plausible that a higher degree of goal attainment should cause more *exaggerating* of positive emotions. However, the issue cannot be resolved in this study.

Third, with 113 participants and about 500 analyzed interactions the sample size is relatively small; furthermore, it is geographically constrained to the French speaking region of Switzerland. Some studies found cross-cultural differences in emotion regulation and its effects (e.g., Grandey et al., 2005b; Fischbach et al., 2006), and this has to be considered. In addition, France and the French part of Switzerland are known to show particularly high scores in power distance, a measure that indicates a particularly low relationship closeness between employes and superiors (Hofstede, 1993), thus, results for other cultures might well differ.

Fourth, when using event-sampling methodology, there are always constraints in the number of questions that can be asked without losing compliance (Nezlek, 1990). We therefore could only ask people if they had a goal but could not ask more specifically about the nature of these goals. The brief descriptions participants gave concerning the interaction sometimes contain hints about possible goals, indicating a wide variety of topics, as one would expect in a work setting (e.g., “I asked my boss if I could leave early”; “Help a client solve a problem”; “No computer in my office”; “Pay raise”). However, these comments were not always informative, and where goals were described we do not know specifics about them (e.g., how large a pay rise the participant expected), nor do we know to which extent the goals were focused on solving a problem (e.g., achieve a solution concerning division of labor) or on one’s personal standing (e.g., not being made responsible for a problem).

Lastly, given the constraint in the length of the study, we did not control for emotional intelligence, and therefore could not investigate how emotional intelligence might influence the link between amplifying and goal attainment. We did control for extraversion and neuroticism, which are strong correlates of trait emotional intelligence (Van der Zee et al., 2002; Petrides et al., 2010). Nevertheless, future studies should include the emotional intelligence measures, especially the dimensions of perceiving and managing emotions (cf. Salovey and Grewal, 2005).

This study also has strengths. First, we investigated effects of emotion expression and display regulation in everyday interactions, and thus can show differences and similarities to experimental research. Second, we particularly focused on the expression and amplification of positive emotions in interactions; most research related to display regulation at work has been done in the context of emotion work with an emphasis on regulating the expression of felt *negative* emotions; this also applies to research that focuses on social interactions (Friedman et al., 2004; Van Kleef and Cote, 2007). Showing that there may be circumstances in which amplifying positive emotions benefits goal attainment therefore constitutes a unique contribution, since simply showing the positive emotion authentically already would likely be associated with considerable benefits but less risk.

Although a vast literature on impression management indicated that a general tendency to amplify positive emotions can lead to general positive outcomes at work, our study contributes to showing where exactly this tactic is used and with what effect; in this sense, it contributes to the impression management literature. Furthermore, our findings also demonstrate how important it is to consider *who* is in the interaction, underscoring the role and the status of interaction partners at work.

### Implications for further research

There are several implications of our results for further research. One issue relates to the type of goals people pursue. As indicated by the short descriptions people gave about the interactions, they do pursue all kinds of task-related goals in interactions. Which type of goals is most frequently pursued by means of expressing positive emotions, however, requires further research that specifies the goals involved. One interesting distinction in this context relates to goals that are related to one’s work (e.g., getting a new computer) versus goals that are related to the person him- or her-self, e.g., appearing competent, dependable, etc., but also avoiding negative outcomes such as being blamed for mistake (cf. Cropanzano et al., 1993). Such goals are implied by the research on impression management, but they should be assessed in greater detail in daily interactions. Note that this type of goal may well be pursued in parallel with task- and job-related goals. Also, it is important to investigate the relative importance of the goals involved. From our research one might conclude that it is relatively easy for employes to “manipulate” their superiors. However, it is conceivable that the goals attained by our participants were not very far-reaching, but rather small-scale, everyday goals without substantial implications for the long-term strategy of the superiors. How far the influence of expressing positive emotions goes in terms of more “strategic” goals is an issue that should be investigated.

### Final remarks

Together, our findings contribute to the existing literature on display regulation of emotions in interactions at work by showing that expressing positive emotions may not only benefit the organization to the detriment of the employe (Hochschild, 1983); rather, display regulation may also help to achieve individual goals, and thus create success experiences, which then benefit the individual (Gross et al., 2011). Whereas authentic display of positive emotions seems to be beneficial for goal attainment throughout, amplifying positive emotions evidently works specifically when interacting with superiors.

## Conflict of Interest Statement

The authors declare that the research was conducted in the absence of any commercial or financial relationships that could be construed as a potential conflict of interest.
